# Mycoplasmas: sophisticated, reemerging, and burdened by their notoriety.

**DOI:** 10.3201/eid0301.970103

**Published:** 1997

**Authors:** J B Baseman, J G Tully

**Affiliations:** Department of Microbiology, University of Texas Health Science Center, San Antonio 78284-7758, USA. baseman@uthscsa.edu

## Abstract

Mycoplasmas are most unusual self-replicating bacteria, possessing very small genomes, lacking cell wall components, requiring cholesterol for membrane function and growth, using UGA codon for tryptophan, passing through "bacterial-retaining" filters, and displaying genetic economy that requires a strict dependence on the host for nutrients and refuge. In addition, many of the mycoplasmas pathogenic for humans and animals possess extraordinary specialized tip organelles that mediate their intimate interaction with eucaryotic cells. This host-adapted survival is achieved through surface parasitism of target cells, acquisition of essential biosynthetic precursors, and in some cases, subsequent entry and survival intracellularly. Misconceptions concerning the role of mycoplasmas in disease pathogenesis can be directly attributed to their biological subtleties and to fundamental deficits in understanding their virulence capabilities. In this review, we highlight the biology and pathogenesis of these procaryotes and provide new evidence that may lead to increased appreciation of their role as human pathogens.


### Mycoplasmas: Sophisticated, Reemerging, and Burdened by Their Notoriety

**Joel B. Baseman* and Joseph G. Tully†**
*The University of Texas Health Science Center at San Antonio, San Antonio, Texas, USA; †National Institute of Allergy and Infectious Diseases, Frederick Cancer Research and Development Center, Frederick, Maryland, USA

*"Sit down before fact as a little child, be prepared to give up every preconceived notion, follow humbly wherever and to whatever abysses nature leads, or you shall learn nothing."*
Thomas Henry Huxley

Mycoplasmas are most unusual self-replicating bacteria, possessing very small genomes, lacking cell wall components, requiring cholesterol for membrane function and growth, using UGA codon for tryptophan, passing through "bacterial-retaining" filters, and displaying genetic economy that requires a strict dependence on the host for nutrients and refuge. In addition, many of the mycoplasmas pathogenic for humans and animals possess extraordinary specialized tip organelles that mediate their intimate interaction with eucaryotic cells. This host-adapted survival is achieved through surface parasitism of target cells, acquisition of essential biosynthetic precursors, and in some cases, subsequent entry and survival intracellularly. Misconceptions concerning the role of mycoplasmas in disease pathogenesis can be directly attributed to their biological subtleties and to fundamental deficits in understanding their virulence capabilities. In this review, we highlight the biology and pathogenesis of these procaryotes and provide new evidence that may lead to increased appreciation of their role as human pathogens.

No other group of procaryotes has been so embroiled in controversy and in establishing a clear pathogenic niche as the mycoplasmas. Their virulence determinants are undeniably complex, and their unique biological properties likely challenge the host differently from typical bacterial pathogens (1,2). Also, numerous *Mycoplasma* species appear to comprise the commensal microbial flora of healthy persons (3), and the association of these mycoplasmas with disease complicates the diagnosis and necessitates extensive and highly specific serologic, nucleic acid, and epidemiologic data. Nonetheless, mycoplasmas by themselves can cause acute and chronic diseases at multiple sites with wide-ranging complications and have been implicated as cofactors in disease. Recently, mycoplasmas have been linked as a cofactor to AIDS pathogenesis and to malignant transformation, chromosomal aberrations, the Gulf War Syndrome, and other unexplained and complex illnesses, including chronic fatigue syndrome, Crohn's disease, and various arthritides (4-8). Even with mounting evidence of their pervasive and pathogenic potential, mycoplasmas still evoke the image of a group of obscure or impotent microorganisms. Yet they are evolutionarily advanced procaryotes (9-11), and their elite status as "next generation" bacterial pathogens necessitates new paradigms in fully understanding their disease potential.

Mycoplasmas, which lack cell walls but possess distinctive sterol-containing plasma membranes, are taxonomically separated from other bacteria and belong to the class Mollicutes (*mollis,* soft; *cutis,* skin). Mollicutes, a term that includes the cell wall-less procaryotes assigned to numerous genera under the class Mollicutes and is frequently used interchangeably with mycoplasmas, are unusual for other biological reasons

Address for correspondence: Joel B. Baseman, Department of Microbiology, The University of Texas Health Science Center at San Antonio, 7703 Floyd Curl Drive, San Antonio, TX 78284-7758; fax: 210-567-6491; e-mail: baseman@uthscsa.edu.

as well. They are evolutionary descendants of the low G+C containing gram-positive bacteria and, through chromosome reduction, represent the smallest self-replicating life forms. Their streamlined genome size, which illustrates extreme biological gene economy, imposes complex nutritional requirements, such as dependence on external supplies of biosynthetic precursors, including amino acids, nucleotides, fatty acids, and sterols. This limited coding capacity dictates for mycoplasmas a parasitic way of life that few pathogenic microorganisms can claim. Therefore, the view that pathogenic mycoplasmas can grow "independently" requires an appreciation of their fastidious nature and their intimate dependence upon the host. Because of these properties, pathogenic mycoplasmas are among the most difficult microorganisms to grow from clinical specimens and remain frequent contaminants of primary and continuous eucaryotic cell lines and tissue cultures (12). In some instances, mycoplasma contamination is obvious since infected eucaryotic cells exhibit aberrant growth, metabolism, and morphology. However, mycoplasmas often establish covert and chronic infections of target cells that lead to either invalid and misleading data or introduction of mycoplasmas or their products into reagents dedicated to therapeutic or research purposes. The recent emphasis on isolating viral agents, such as human immunodeficiency virus (HIV)-1, from human primary lymphocytic cells has also demonstrated the frequent cocultivation of mycoplasmas of human origin. Often, the unwanted sources of exogenous mycoplasmas are serum products and filter-"sterilized" (450 nm) solutions; cross-contamination by already infected cell cultures, viral stocks, or immunologic preparations; breaks in technique, including aerosols from the respiratory tract or by mouth pipetting; ignorance of the mycoplasma problem; or scientific indifference.

Detailed up-to-date reviews describing the biological and pathogenic properties of mycoplasmas have been published (1,2,13,14). Our intention here is to provide a concise historical perspective of the role of mycoplasmas in human disease; highlight the discoveries of new *Mycoplasma* species and their association with human illness and host conditions that present problems in detection and treatment; describe selected biological properties of mycoplasmas consistent with their intimate host relationship and possible mechanisms of pathogenicity; and address recent controversies associated with mycoplasmas as emerging infectious agents. Renewed attention to these issues may provide the impetus to demystify mycoplasmas and improve their standing as genuine, card-carrying pathogens.

### Historical Perspectives

The earliest reports of mycoplasmas as infectious agents in humans appeared in the 1930s and 1940s. At that time, primary atypical pneumonia was associated with an infectious agent that because of its minute size and innate biological properties unknown at that time, passed through bacteria-retaining filters, resisted penicillin and sulfonamide therapies, and adapted to growth in embryonated eggs and tissue culture cells. Correlations between the etiologic agent of "walking pneumonia" with viruses, L-forms, and pleuropneumonialike agents (referred to as PPLOs in publications and textbooks of that era) were frequent and often misleading. Finally, definitive studies in the early 1960s established *Mycoplasma pneumoniae* as the singular cause of cold agglutinin-associated primary atypical pneumonia (2). Today *M. pneumoniae* remains an important cause of pneumonia and other airway disorders, such as tracheobronchitis and pharyngitis (13,14), and is associated with extrapulmonary manifestations, such as hematopoietic, exanthematic, joint, central nervous system, liver, pancreas, and cardiovascular syndromes (15).

The confusion associated with *M. pneumoniae*-mediated infections has recurred many times with other mycoplasmas, whose detection in clinical specimens through culture, antibody, or DNA-based testing is frequently dismissed as "only mycoplasmas" even when they appear to be the primary pathogens. Two mycoplasmas commonly found in the urogenital tracts of healthy persons are *Mycoplasma hominis* and *Ureaplasma urealyticum*. However, over the years, the pathogenic roles of these mycoplasmas have been proven in adult urogenital tract diseases, neonatal respiratory infections, and a range of other diseases usually in immunocompromised patients (2).

Several recent examples illustrate the increasing impact of *Mycoplasma* species on emerging diseases. *Mycoplasma fermentans* strains were first isolated from the lower genital tract of both adult men and women in the early 1950s, but their role in classic lower genital tract disease has not been established (16). Reports in the 1970s of *M. fermentans* in the joints of rheumatoid arthritis patients and in the bone marrow of children with leukemia raised expectations for its pathogenic potential (17,18); these findings have not been adequately confirmed. Sufficient evidence, however, has accumulated recently to establish an important and emerging role for *M. fermentans* in human respiratory and joint diseases. For example, *M. fermentans* has been detected by specific gene amplification techniques such as polymerase chain reaction (PCR) in the synovial fluid of patients with inflammatory arthritis, but not in the joints of patients with juvenile or reactive arthritis (19). In two other studies using PCR, *M. fermentans* was identified in the upper respiratory tract of 20% to 44% of both healthy and HIV-infected patients (20,21) and was associated with acute respiratory distress syndrome in nonimmunocompromised persons (22).

*Mycoplasma genitalium* was detected in the urogenital tract of two patients with nongonococcal urethritis in 1981 (23), but for more than a decade, very little was known about its host distribution and pathogenicity. Early experimental studies established that the organism caused lower genital tract infections in both male and female chimpanzees, with extensive urethral colonization in males and apparent tissue invasion, eventually leading to overt bacteremia (24). However, the fastidious growth requirements of *M. genitalium* from human hosts severely limited further study until the advent of molecular detection techniques. Specific sequences in the 140 kDa adhesin protein gene of *M. genitalium* were selected as targets in a PCR-based detection assay (25,26). Subsequent application of these techniques in cases of acute nongonococcal urethritis, not including those of patients colonized or infected with *Chlamydia trachomatis,* has provided mounting evidence for the involvement of *M. genitalium* as an etiologic agent of this disease (27-29). Also, *M. genitalium* has been suspected in chronic nongonococcal urethritis and pelvic inflammatory disease (30).

The discovery in 1988 of *M. genitalium* strains in human nasopharyngeal throat specimens, where they were frequently mixed with strains of *M. pneumoniae,* not only changed dramatically the concept of host distribution of *M. genitalium* but also prompted critical questions about the role of this mycoplasma in human respiratory disease (31). However, the immunologic cross-reactivity between *M. genitalium* and *M. pneumoniae* and the inability of most conventional diagnostic serologic tests to conclusively identify *M. genitalium* have complicated its delineation in acute human respiratory disease. PCR assays specific for the organism have detected *M. genitalium* in throat specimens of patients infected with HIV-1 (32). However, these probes have not been applied to control groups and patients in outbreaks of acute respiratory disease and/or pneumonia to determine whether *M. genitalium* alone is an etiologic agent in respiratory infections.

*M. genitalium* has been implicated as an etiologic agent in certain human joint diseases. This clinical correlation began with the observation of a mixed infection of *M. pneumoniae* and *M. genitalium* in synovial fluid specimens of a nonimmunocompromised patient after an acute respiratory infection (33). A predominant role was not established for either *Mycoplasma* species in the initial respiratory disease or in the joint manifestations, although evidence to implicate postinfectious autoimmunity to both organisms was described. These findings prompted a PCR assay on synovial fluids from patients with various arthritic syndromes, which presented case reports on two of 13 patients with *M. genitalium* detected in joint fluids (34).

Another area of emerging mycoplasmal infections concerns immunodeficiency. Although patients with congenital or acquired disorders of antibody production are susceptible to a wide variety of microbial infections, the unique susceptibility of such patients to mycoplasmal infections is a growing concern, especially considering the number of occurrences, the types of mycoplasmas involved, and the difficulties posed in the therapeutic management of such infections. In addition, the increased use of prolonged or permanent immunosuppressive chemotherapy required for patients undergoing tissue or organ transplantation or treatment of various malignant diseases has also increased the risk for mycoplasmal infections—from mycoplasmas that are part of the normal human mollicute flora to those acquired through animal contact.

The association between immunodeficiency and mycoplasmal infections was first reported in the mid 1970s in patients with primary hypogammaglobulinemia and infection with *U. urealyticum*, *M. pneumoniae*, *Mycoplasma salivarium*, and *M. hominis* that localized in joint tissue, frequently with destructive arthritis. Similar joint infections in hypogammaglobulinemic patients with these mycoplasmal species continue to be reported (35). Since most of these mollicutes, with the possible exception of *M. pneumoniae*, occur as part of the normal human flora, the origin of such joint infections is considered endogenous. Patients with hypogammaglobulinemia and other antibody deficiencies are also especially susceptible to mycoplasmal infections of the upper respiratory and urinary tracts caused most frequently by *M. pneumoniae* or *U. urealyticum*, respectively (36).

Mycoplasmal infections following organ transplantation and immunosuppressive chemotherapy were observed in the early 1980s, with both *M. hominis* and *U. urealyticum* reported most often (37-39). Although these infections most likely originated from the patient's normal microbial flora, a recent report of donor transmission of *M. hominis* to two lung allograft recipients (40) suggests that donor tissue may be a more important factor in transplant infections than currently recognized.

While patients with antibody defects or those receiving immunosuppressive drugs appear to be the most susceptible to infections with mycoplasmas present in healthy tissues, emerging evidence indicates that contact with other mycoplasmas in the environment is an important hazard. For example, the direct isolation of a feline mycoplasma (*M. felis*) from the joint of a hypogammaglobulinemic patient with septic arthritis was recently reported (41), with suspected transmission occurring through a cat bite 6 months before the onset of arthritis. Other examples include fatal septicemia caused by *M. arginini*, a common animal mycoplasma, from blood and multiple tissue sites in a slaughter house employee who had advanced non-Hodgkin's lymphoma and hypogammaglobulinemia (42), and a septicemic infection with a canine mycoplasma (*M. edwardii*) in a patient with advanced AIDS (M.K. York, pers. comm.).

One of the most critical aspects of mycoplasmal infections in immunodeficient patients is the frequent inability to control such infections with appropriate broad spectrum antibiotics. Although the tetracyclines and erythromycins are effective chemotherapeutic agents for many mycoplasmal infections, *M. fermentans* and *M. hominis* strains are usually resistant to erythromycin, and tetracycline-resistant strains of *M. hominis* and *U. urealyticum* have been reported from the lower urogenital tract of patients. However, these antibiotics and most other broad spectrum agents have limited mycoplasmacidal activity in vivo, and their efficacy eventually depends on an intact host immune system to eliminate the mycoplasmas. Most hypogammaglobulinemic patients lack the ability to mount a strong antibody response. Guidelines for managing such mycoplasmal infections in patients with immune defects should include immediate in vitro testing of the isolated mollicute against a wide range of antibiotics; expeditious administration of the antibiotic by the most appropriate route (intravenously, if warranted); prolonged therapy terminated only if there is no rapid clinical or microbiological response; and possibly administration of intravenous immunoglobulin (35,36). Clinical management of mycoplasmal infections in transplant patients is more difficult since immunoglobulins may enhance graft or organ rejection. In the absence of suitable mycoplasmacidal chemotherapeutic agents, vigorous and sustained chemotherapy with the most active antibiotic is the current method of choice.

### Mechanisms of Pathogenicity

Many mycoplasmal pathogens exhibit filamentous or flask-shaped appearances and display prominent and specialized polar tip organelles that mediate attachment to host target cells (43,44). These tip structures are complex, composed of a network of interactive proteins, designated adhesins, and adherence-accessory proteins (Figure 1, [14,43]). These proteins cooperate structurally and functionally to mobilize and concentrate adhesins at the tip and permit mycoplasmal colonization of mucous membranes and eucaryotic cell surfaces, probably through host sialoglycoconjugates and sulfated glycolipids (Figure 2, [14,43,45]). It appears that mycoplasmal cytadherence-related proteins represent a superfamily of genes and proteins that have been conserved through horizontal gene transfer from an ancestral gene family. This protein network resembles a specialized cytoskeletonlike apparatus, which may represent the precursor to mammalian cytoskeletal and extracellular matrixlike complexes (14). Other *Mycoplasma* species lack distinct tip structures yet are capable of cytadherence, and they may use related genes or proteins or alternative mechanisms of surface parasitism.

The family of mycoplasmal genes and proteins involved in cytadherence has been studied most extensively in *M. pneumoniae* (14,43,46-48). Noncytadhering phenotypes that arise through spontaneous mutation at high frequency have been categorized into mutant classes on the basis

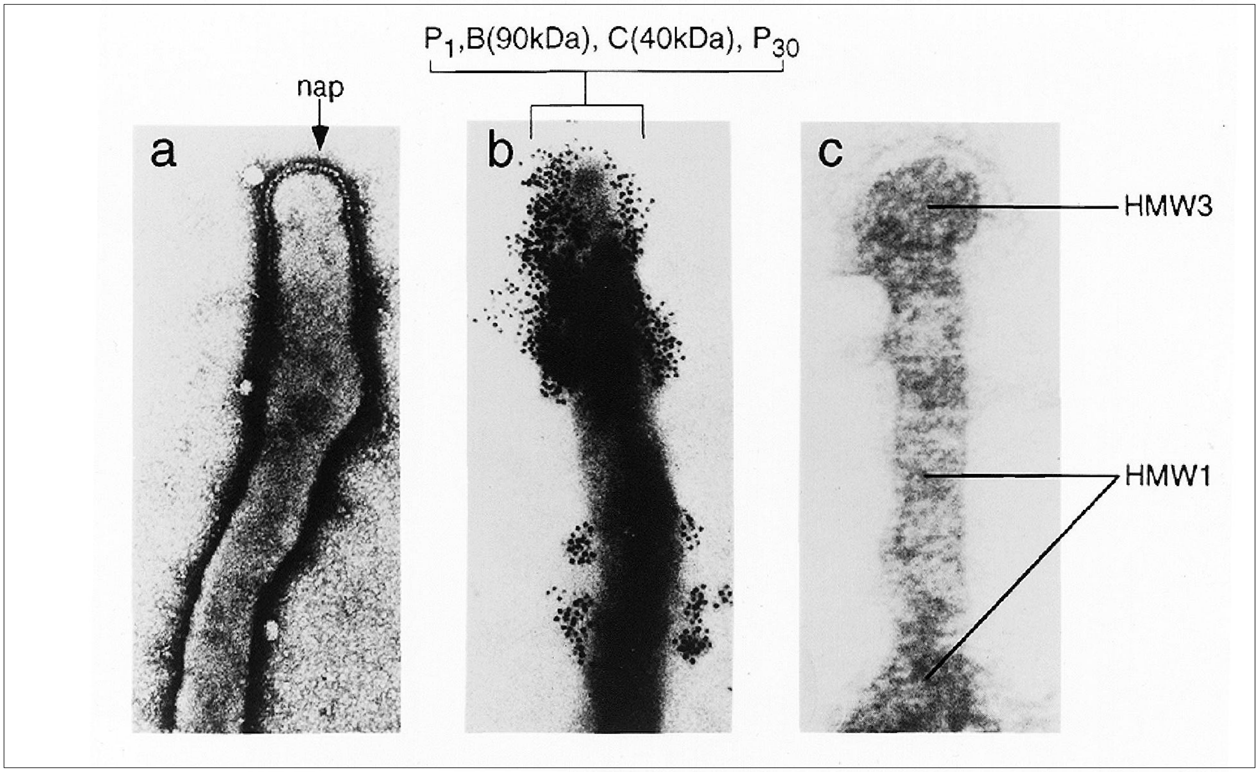

Figure 1. Transmission electron photomicrographs of the specialized tip organelle of cytadherence-positive *M. pneumoniae* demonstrating a) truncated structure with nap, b) clustering of cytadherence-related proteins (P1, B, C, P30) at the tip based on immunolabeling with ferritin and colloidal gold and crosslinking studies, and c) Triton X-100–resistant, cytoskeleton-like, structure with distinct bleb and parallel filaments (14,43,45,46).

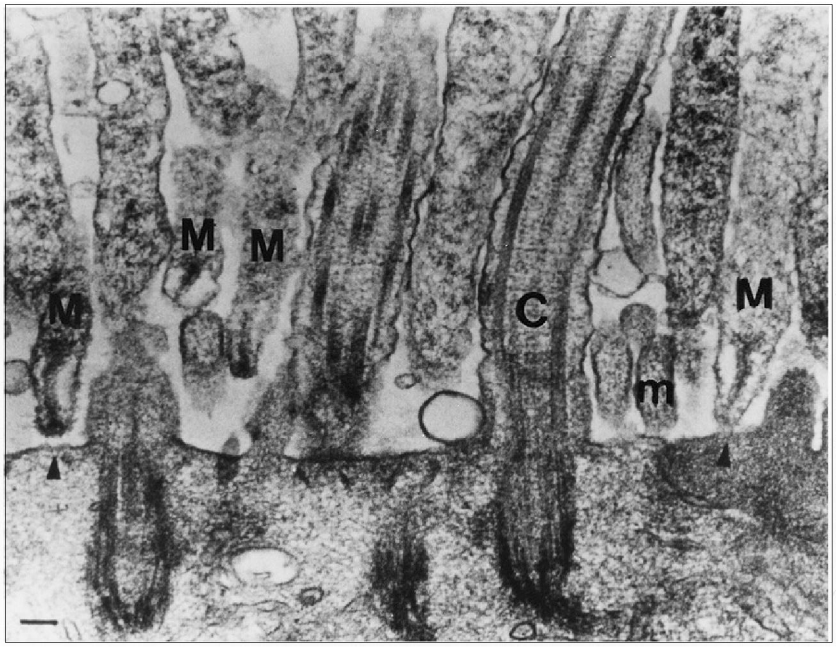

Figure 2. Transmission electron photomicrograph of a hamster trachea ring infected with *M. pneumoniae* (43). Note the orientation of the mycoplasmas through their specialized tiplike organelle, which permits close association with the respiratory epithelium. M, mycoplasma; m, microvillus; C, cilia.

of distinct protein profiles. These noncytadhering mycoplasmas cannot synthesize specific cytadherence-related proteins or are unable to stabilize them at the tip organelle, which leads to abnormal anatomical tip structures and avirulence (43). Spontaneous reversion to the cytadhering phenotype is accompanied by the reappearance of the implicated proteins, restoration of structurally and functionally intact tips, and return of full infectivity (43). Similar cytadherence-related genes and proteins have been reported for *M. genitalium* on the basis of biochemical, immunologic, and genetic analyses (25,49,50). Furthermore, striking similarities exist in the order of operons that comprise the cytadherence-related genes and the organization of these genes within each operon of *M. pneumoniae* and *M. genitalium* (14,50,51). These similarities reinforce the unexpected coisolations of *M. genitalium*, along with *M. pneumoniae*, from the nasopharyngeal throat swabs of patients with acute respiratory diseases and from synovial fluids of patients with arthritis as described in the previous section (31,33). The isolation of *M. pneumoniae* from the human urogenital tract (52) further suggests that these mycoplasmas have evolved parasitic strategies that include overlapping tissue tropisms as determined by the genetic and chemical relatedness of their cytadherence genes and proteins (14,25,43,50,51). The recent use of transposon mutagenesis to generate *M. pneumoniae* and *M. genitalium* transformants displaying cytadherence-deficient phenotypes should further clarify the relationships between the cytadherence-related genes and proteins and identify additional sites previously unlinked to cytadherence (46,53).

An interesting feature of specific *M. pneumoniae* and *M. genitalium* adhesins is their multiple gene copy nature (14,43,54,55,56). Although only one full-length copy of the adhesin structural genes exists in adhesin-related operons, precise regions of these adhesin genes are detected as single genomic copies, while other regions occur as closely homologous, but not identical, multiple copies. In other words, multiple truncated and sequence-related copies of the adhesin genes are dispersed throughout the genome, which could generate adhesin variation through homologous recombination. Consistent with this possibility is the existence of restriction fragment length polymorphisms in the adhesin genes of human clinical isolates of *M. pneumoniae* and *M. genitalium*, reflected by sequence divergence in the multiple-copy regions of the adhesin genes (56-59). It appears that a repertoire of partial adhesin-related gene regions serves as a reservoir to regulate the structural and functional properties of mycoplasmal adhesins through recombination events, which may lead to circumvention of the host immune response. Mechanisms of phase and antigenic variation are likely to occur in which mycoplasmal adhesins exhibit altered specificities and affinities, as determined by the organization of constant and variable adhesin gene sequences. Therefore, despite their small genomes, pathogenic mycoplasmas facilitate DNA rearrangements through repetitive gene sequences, thus promoting genetic diversity and maximizing the coding potential of their limited genomes. The immunodominant epitopes of the mycoplasmal adhesins appear not to be identical to the adherence-mediating domains (13). The latter are in part encoded by single copy regions of the adhesin genes and are highly conserved, which reinforces their essential role in mycoplasmal recognition of host cell receptors and colonization (60,61). Host immunoresponsiveness directed at the noncytadherence-mediating variable regions is unlikely to generate effective cytadherence-blocking antibodies, which may in part clarify the observed high reinfection rates of patients. Thus, the grouping of clinical isolates of *M. pneumoniae* into two categories, on the basis of sequence divergence in the multiple-copy regions of the adhesin gene (56-59), along with the immune status of the population, may explain the epidemiologic patterns of *M. pneumoniae* reported over the years.

Another characteristic of the cytadherence-related proteins is their proline-rich composition, which markedly influences protein folding and binding. Several reports have established the importance of these proline-rich domains in mycoplasmal cytadherence and virulence (47,48,62,63), and recent evidence further suggests that mycoplasmal peptidyl-prolyl isomerases, i.e., cyclophilins, are critical in regulating the conformation and function of the mycoplasmal cytadherence-related tip organelle, colony morphology, and growth (14,64). In addition to this proline-rich property, one of the most unusual features of the adhesins is their extensive sequence homology to mammalian structural proteins (1,14,33,43,47,48). This molecular mimicry is especially interesting since it has been suggested for decades that mycoplasmas provoke an anti-self response that triggers immune disorders, although the basis for the induction has been elusive (65). Patients with documented *M. pneumoniae* respiratory infections demonstrate seroconversion to myosin, keratin, and fibrinogen (33) and exhibit extrapulmonary manifestations, such as exanthems and cardiac abnormalities. Furthermore, a classic example of bacteria-mediated autoimmune disorders is the development of acute rheumatic fever following streptococcal infection (66). Anti-streptococcal antibodies reactive against α-helical coiled-coil regions of the M protein cross-react with heart myosin, tropomyosin, and mycoplasmal adhesins (14,66). In the latter case, these mycoplasmal adhesins exhibit amino acid sequence homologies with human CD4 and class II major histocompatibility complex lymphocyte proteins, which could generate autoreactive antibodies and trigger cell killing and immunosuppression (67,68). Also, mycoplasmas may serve as B-cell and T-cell mitogens and induce autoimmune disease through the activation of anti-self T cells or polyclonal B cells. The multiorgan protean manifestations of mycoplasmal infections in humans are consistent with the pathogenesis of autoimmunity. Furthermore, the ability of mycoplasmas to induce a broad range of immunoregulatory events, mediated by cytokine production and direct effects on macrophages, B and T cells, and glial cells, is evidence that mycoplasmas possess the attributes of primary mediators of pathogenesis (1,2,12,69). For example, cytokine production and lymphocyte activation may either minimize disease through the activation of host defense mechanisms or exacerbate disease through lesion development (69,70). Also, a superantigen derived from *Mycoplasma arthritidis*, a mycoplasma pathogenic for rodents, induces arthritis and chronic disease manifestations (69). It has been suggested that related superantigen-like molecules may exist in mycoplasmas of human origin triggering autoimmune and other inflammatory pathologies.

It appears that cytadherence is the initial step in the virulence process of pathogenic mycoplasmas (Figure 2) and precedes a spectrum of subtle or overt host cell responses. In specific instances, distinct cytopathology correlates with the infecting *Mycoplasma* species, the number of adherent mycoplasmas, the length of coincubation, the induction of proinflammatory cytokines, and the age and immune status of the patient. For example, the exacerbation of clinical syndromes may correlate with a history of mycoplasmal infection as observed in patients with recurrent *M. pneumoniae* exposures (2,13). Also, the elevated expression of proinflammatory cytokines associated with mycoplasmal disease pathogenesis may coincide with the intensity of the symptoms. In other cases, chronic disease or no obvious signs or symptoms of disease accompany mycoplasmal infection.

Other biological properties of mycoplasmas have been implicated as virulence determinants and include 1) generation of hydrogen peroxide and superoxide radicals by adhering mycoplasmas, which induces oxidative stress, including host cell membrane damage; 2) competition for and depletion of nutrients or biosynthetic precursors by mycoplasmas, which disrupts host cell maintenance and function; 3) existence of capsule-like material and electron-dense surface layers or structures, which provides increased integrity to the mycoplasma surface and confers immunoregulatory activities; 4) high-frequency phase and antigenic variation, which results in surface diversity and possible avoidance of protective host immune defenses; 5) secretion or introduction of mycoplasmal enzymes, such as phospholipases, ATPases, hemolysins, proteases, and nucleases into the host cell milieu, which leads to localized tissue disruption and disorganization and chromosomal aberrations; and 6) intracellular residence, which sequesters mycoplasmas, establishes latent or chronic states, and circumvents mycoplasmicidal immune mechanisms and selective drug therapies (1,2,71,72). Whether pathogenic mycoplasmas enter and survive within mammalian cells has been debated for many years. Consistent with this possibility, mycoplasmas exhibit limited biosynthetic capabilities; are highly fastidious and dependent upon the host microenvironment and complex culture medium for growth; have been observed in intimate contact with mammalian cell surfaces and within target cells; may be capable of initiating fusion with host cells through their cholesterol-containing unit membranes; and survive long-term recommended antimicrobial treatment in humans and tissue cultures. Recent sightings of intact mycoplasmas throughout the cytoplasm and the perinuclear regions of tissue cells from infected patients and in cell cultures, along with evidence that mycoplasmas are capable of long-term intracellular survival and replication in vitro, offer an additional dimension to the pathogenic potential of mycoplasmas (4,14,72,73).

### The Latest Controversies: Food for Thought or the Twilight Zone

On the basis of the above information, the virulence strategies displayed by mycoplasmas are likely the summation of a multitude of biological activities (1). Since no obvious single or group of mycoplasmal properties inextricably correlates with disease manifestations, the proof that mycoplasmas are card-carrying pathogens necessitates thorough and highly specific microbiological, epidemiologic, and diagnostic criteria; detailed descriptions of biochemical, genetic, and immunologic characteristics that distinguish virulent and avirulent mycoplasmas; and reproducibility of the symptoms of disease in experimental animal models or in the natural spread of infection among susceptible populations. The portfolio of available evidence concerning mycoplasma-mediated disease pathogenesis is limited. These scientific shortcomings precipitate misconceptions concerning mycoplasmas as singular agents of infectious diseases, as putative cofactors in the progression of other diseases, and as universal contaminants of cell cultures. Clearly, multiple pathways of interaction with target cells appears to be the *modus operandi* of the *Mycoplasma* species. With this conceptual scientific framework as a background, five recently proposed and controversial associations of mycoplasmas to human diseases are worth noting.

### AIDS

The role of mycoplasmas in accelerating the progression of AIDS could not have begun under more baffling and circuitous conditions. A viruslike agent that arose through transfection of NIH 3T3 cells with DNA from Kaposi sarcoma tissues of AIDS patients was later shown to be *M. fermentans*. The spotted history of *M. fermentans* in rheumatoid arthritis and leukemia and its frequent contamination of cell cultures, along with its contemporary link to AIDS, have been considerable impediments to overcome in its elevation to pathogenic status. However, careful and convincing independent studies by several laboratories have implicated *M. fermentans* as a cause of systemic infections and organ failure in AIDS patients (4,74). The isolation of *M. fermentans* from blood and urine samples of HIV-infected persons, its detection by PCR and immunohistochemistry in multiple tissue sites at various stages of AIDS, and its ability to stimulate CD4+ lymphocytes and other immunomodulatory activities implicate this *Mycoplasma* species as a cofactor in AIDS. Consistent with this possibility, *M. fermentans* has been shown to act synergistically with HIV to enhance cytopathic effects on human CD4+ lymphocytes. Coincident with these studies, a new *Mycoplasma* species, *Mycoplasma penetrans*, also has emerged as a potential cofactor in AIDS progression (75,76). Its isolation almost exclusively from the urine of HIV-infected patients, the extraordinarily high prevalence of antibodies against this mycoplasma in HIV-infected patients and not in HIV-seronegative persons, and its capacity to invade target cells and activate the immune system of HIV-infected patients at various stages of disease correlate with a synergistic role with HIV. Other mycoplasmas, including *M. genitalium* and *Mycoplasma pirum*, have also been isolated from AIDS patients and implicated as potential cofactors. However, the proposed role of mycoplasmas as infectious agents and cofactors in AIDS-related disorders still remains a hypothesis without definitive proof. If cofactors of HIV are essential to the development of late stages of HIV-mediated disease, mycoplasmas possess all the prerequisite properties of the consummate helper. Their ability to establish covert or overt chronic and persistent infections with concomitant activation of the immune system, stimulation of cytokine production, and induction of oxidative stress correlate with increased HIV replication and disease progression. Are mycoplasmas irrelevant to AIDS, or are the clinical and microbiological correlations sufficient to imply intimate relationships between HIV and mycoplasmas, especially as the infected host undergoes immunologic distress?

### Malignant Transformation

As early as the mid-1960s, mycoplasma-infected cell lines were associated with chromosomal aberrations, altered morphologies, and cell transformation (77,78). These abnormal oncogenic cell traits continued even after the apparent elimination of mycoplasmas, and evidence implied increased tumorigenicity of these transformed cells in animals. This issue has been revisited in studies demonstrating that long-term, persistent mycoplasmal infection of mouse embryo cells initiated a multistage cellular process that resulted in irreversible cell transformation, karyotypic alterations, and tumorigenicity in nude mice (6). Do these oncogenic events associated with mycoplasma-mammalian cell coincubation relate to the ontogeny of human cancers?

### Gulf War Syndrome

One of the most controversial current medical issues is whether the multiple acute and chronic symptoms found in veterans of the Persian Gulf War were caused by chemical exposure, infectious agents, or psychological problems, or whether a Gulf War Syndrome exists at all. The clinical illness comprises a collection of symptoms, including chronic fatigue, joint pain, headaches, and skin rashes. One study suggests that pathogenic mycoplasmas are responsible for a large number of cases among veterans, on the basis of DNA hybridization and the responsiveness of veterans to prolonged antibiotic treatment (5). Even though the experimental evidence is sparse and incomplete and well-controlled and detailed studies by independent laboratories are needed, if the Gulf War Syndrome has infectious causes, mycoplasmas with their requisite biological credentials are potential candidates.

### Crohn's Disease

Several epidemiologic studies correlate respiratory infections with exacerbation of Crohn's disease and other chronic inflammatory bowel diseases (7,79). Acute onset gastrointestinal symptoms in patients with these diseases are accompanied by seroconversion to specific viral or *M. pneumoniae* antigens. As indicated earlier, mycoplasmas can elicit pleiotropic immune responses and are difficult to eliminate in patients despite appropriate antibiotic treatment. Steroid therapy to control gastrointestinal symptoms in these patients, along with the multifaceted biological properties associated with pathogenic mycoplasmas, may precipitate the onset of acute exacerbations of chronic inflammatory bowel disease.

### Rheumatoid Arthritis and Other Human Arthritides

The occurrence of various *Mycoplasma* and *Ureaplasma* species in joint tissues of patients with rheumatoid arthritis, sexually transmitted reactive arthritis, and other human arthritides can no longer be ignored (8). A clinical trial of long-term (6 to 12 months) antibiotic (doxycycline) therapy before cartilage destruction might prove beneficial in managing such frequent and often debilitating infections.

Extensive clinical and microbiological evidence indicates that mycoplasmas alone can elicit a spectrum of illness for which no other agents are incriminated. The eradication of these pathogenic mycoplasmas from various tissue sites requires an intact and functional immune system, although persons with fully competent immune systems may have difficulty eliminating mycoplasmas, even with recommended prolonged drug therapy. Nonetheless, mycoplasmas are still viewed as subordinates to other infectious agents and are relegated to a category of commensals that unwittingly cause disease in patients whose immune systems offer little resistance to microbial stress and overload.

The fundamental importance of mycoplasmas in specific diseases of humans, animals, insects, and plants is irrefutable, and their unique biological properties are consistent with their intimate association with host target cells. These remarkable bacteria must continue to receive the scientific attention of mycoplasmologists, cell culturists, clinicians, immunologists, and DNA sequencers who most recently are compiling extensive databases that may eventually dissect every approachable mycoplasmal element that defines their biological and genetic being. Nonetheless, mycoplasmas remain mysterious and enigmatic, and the available data and proposed hypotheses that correlate mycoplasmas with disease pathogenesis range from definitive, provocative, and titillating to inconclusive, confusing, and heretical. Controversy seems to be a recurrent companion of mycoplasmas, yet good science and open-mindedness should overcome the legacy that has burdened them for decades.

### Acknowledgments

This study was supported in part by NIH grants AI 27873, AI 32829 and AI 41010.

Dr. Baseman is professor and chair, Department of Microbiology, University of Texas Health Science Center, San Antonio. His research focuses on pathogen-host cell interactions with special interest in defining the biology and virulence determinants of mycoplasmas pathogenic for humans.

Dr. Tully heads the Mycoplasma Section, Laboratory of Molecular Microbiology, National Institute of Allergy and Infectious Diseases, Frederick, Maryland. His interest covers the host distribution, pathogenicity, and taxonomy of mollicutes.

### References

1. Tryon VV, Baseman JB. Pathogenic determinants and mechanisms. In: Maniloff J, McElhaney RN, Finch LR, Baseman JB, editors. Mycoplasmas: molecular biology and pathogenesis. Washington (DC): American Society for Microbiology, 1992:457-71.
2. Krause DC, Taylor-Robinson D. Mycoplasmas which infect humans. In: Maniloff J, McElhaney RN, Finch LR, Baseman JB, editors. Mycoplasmas: molecular biology and pathogenesis. Washington (DC): American Society for Microbiology, 1992:417-44.
3. Tully JG. Current status of the mollicute flora of humans. Clin Infect Dis 1993;17:S2-9.
4. Lo S-C. Mycoplasmas and AIDS. In: Maniloff J, McElhaney RN, Finch LR, Baseman JB, editors. Mycoplasmas: molecular biology and pathogenesis. Washington (DC): American Society for Microbiology, 1992:525-45.
5. Nicolson G, Nicolson NL. Diagnosis and treatment of mycoplasmal infections in Gulf War illness-CFIDS patients. Intl J Occup Med Immunol Toxicol 1996;5:69-78.
6. Tsai S, Wear DJ, Shih JW-K, Lo SC. Mycoplasmas and oncogenesis: persistent infection and multistage malignant transformation. Proc Natl Acad Sci USA 1995;92:10197-201.
7. Ekbom A, Daszak P, Kraaz W, Wakefield AJ. Crohn's disease after in-utero measles virus exposure. Lancet 1996;348:516-7.
8. Taylor-Robinson D. Mycoplasmas in rheumatoid arthritis and other human arthritides. J Clin Pathol 1996;49:781-2.
9. Bové JM. Molecular features of mollicutes. Clin Infect Dis 1993;17:S10-31.
10. Razin S. Molecular properties of mollicutes: a synopsis. In: Razin S, Tully JG, editors. Molecular and diagnostic procedures in mycoplasmology, Vol I. New York: Academic Press, Inc., 1995:1-25.
11. Dybvig K, Voelker LL. Molecular biology of mycoplasmas. Annu Rev Microbiol 1996;50:25-57.
12. McGarrity GJ, Kotani H, Butler GH. Mycoplasmas and tissue culture cells. In: Maniloff J, McElhaney RN, Finch LR, Baseman JB, editors. Mycoplasmas: molecular biology and pathogenesis. Washington (DC): American Society for Microbiology, 1992:445-54.
13. Jacobs E. *Mycoplasma pneumoniae* virulence factors and the immune response. Rev Med Microbiol 1991;2:83-90.
14. Baseman JB, Reddy SP, Dallo SF. Interplay between mycoplasma surface proteins, airway cells, and the protean manifestations of mycoplasma-mediated human infections. Am J Respir Crit Care Med 1996;154:S137-44.
15. Murray HW, Masur H, Senterfit LB, Roberts RB. The protean manifestations of *Mycoplasma pneumoniae* infection in adults. Am J Med 1975;58:229-42.
16. Deguchi T, Gilroy CB, Taylor-Robinson D. Failure to detect *Mycoplasma fermentans*, *Mycoplasma penetrans*, or *Mycoplasma pirum* in the urethra of patients with acute non-gonococcal urethritis. Eur J Clin Microbiol Infect Dis 1996;15:169-71.
17. Williams MH, Brostoff J, Roitt IM. Possible role of *Mycoplasma fermentans* in pathogenesis of rheumatoid arthritis. Lancet 1970;ii:270-80.
18. Murphy WH, Gullis C, Dabich L, Heyn R, Zarafonetis CJD. Isolation of *Mycoplasma* from leukemic and nonleukemia patients. J Nat Cancer Inst 1970;45:243-51.
19. Schaeverbeke T, Gilroy CB, Bébéar C, Dehais J, Taylor-Robinson D. *Mycoplasma fermentans*, but not *M. penetrans*, detected by PCR assays in synovium from patients with rheumatoid arthritis and other rheumatic disorders. J Clin Pathol 1996;41:311-4.
20. Katseni VL, Gilroy CB, Ryait BK, Ariyoshi K, Bieniasz PB, Weber JN, et al. *Mycoplasma fermentans* in individuals seropositive and seronegative for HIV-1. Lancet 1993;341:271-3.
21. Chingbingyong MI, Hughes CV. Detection of *Mycoplasma fermentans* in human saliva with a polymerase chain reaction-based assay. Archs Oral Biol 1996;41:311-4.
22. Lo SC, Dawson MS, Newton III PB, Sonoda MA, Shih JW, Engler WF, et al. Association of the virus-like infectious agent originally reported in patients with AIDS with acute fatal disease in previously healthy non-AIDS patients. Amer J Trop Med Hyg 1989;41:364-76.
23. Tully JG, Taylor-Robinson D, Cole RM, Rose DL. A newly discovered mycoplasma in the human urogenital tract. Lancet 1981;i:1288-91.
24. Tully JG, Taylor-Robinson D, Rose DL, Furr PM, Graham CE, Barile MF. Urogenital challenge of primate species with *Mycoplasma genitalium* and characteristics of infection induced in chimpanzees. J Infect Dis 1986;153:1046-54.
25. Dallo SF, Chavoya A, Su CJ, Baseman JB. DNA and protein sequence homologies detected between the adhesins of *Mycoplasma genitalium* and *Mycoplasma pneumoniae*. Infect Immun 1989;57:1059-65.
26. Palmer HM, Gilroy CB, Thomas BJ, Naidoo ROM, Taylor-Robinson D. Development and evaluation of the polymerase chain reaction to detect *Mycoplasma genitalium*. FEMS Microbiol Lett 1991;77:199-204.
27. Horner PJ, Gilroy CB, Thomas BJ, Naidoo ROM, Taylor-Robinson D. Association of *Mycoplasma genitalium* with acute non-gonococcal urethritis. Lancet 1993;342:582-5.
28. Deguichi T, Komeda H, Yasuda M, Tada K, Iwata H, Asano M, et al. *Mycoplasma genitalium* in nongonococcal urethritis. Int J STD & AIDS 1995;6:144-6.
29. Jensen SJ, Hansen HT, Lind K. Isolation of *Mycoplasma genitalium* strains from the male urethra. J Clin Microbiol 1996;34:286-91.
30. Taylor-Robinson D. The history and role of *Mycoplasma genitalium* in sexually transmitted diseases. Genitourin Med 1995;71:1-8.
31. Baseman JB, Dallo SF, Tully JG, Rose DL. Isolation and characterization of *Mycoplasma genitalium* strains from the human respiratory tract. J Clin Microbiol 1988;26:2266-9.

32. De Barbeyrac B, Bernet-Poggi C, Febrer F, Renaudin H, Dupon M, Bebear C. Detection of *Mycoplasma pneumoniae* and *Mycoplasma genitalium* in clinical samples by polymerase chain reaction. Clin Infect Dis 1993;17(Supp1):S83-9.
33. Tully JG, Rose DL, Baseman JB, Dallo SF, Lazzell AL, Davis CP. *Mycoplasma pneumoniae* and *Mycoplasma genitalium* mixture in synovial fluid isolate. J Clin Microbiol 1995;33:1851-5.
34. Taylor-Robinson D, Gilroy CB, Horowitz S, Horowitz J. *Mycoplasma genitalium* in the joints of two patients with arthritis. Eur J Clin Microbiol Infect Dis 1994;13:1066-9.
35. Furr PM, Taylor-Robinson D, Webster ADB. Mycoplasmas and ureaplasmas in patients with hypogammaglobulinemia and their role in arthritis: microbiological observations over twenty years. Ann Rheum Dis 1994;53:183-7.
36. Gelfand EW. Unique susceptibility of patients with antibody deficiency to *Mycoplasma* infection. Clin Infect Dis 1993;17:S250-3.
37. Mokhbat JE, Person PK, Sabath LD, Robertson JA. Peritonitis due to *Mycoplasma hominis* in a renal transplant patient. J Infect Dis 1982;146:713.
38. Burdge DR, Reid GD, Reeve CE, Robertson JA, Stemke GW, Bowie WR. Septic arthritis due to dual infection with *Mycoplasma hominis* and *Ureaplasma urealyticum*. J Rheumatol 1988;15:366-8.
39. Luttrell LM, Kanj SS, Corey GR, Lins RE, Spinner RJ, Mallon WJ, et al. *Mycoplasma hominis* septic arthritis: two case reports and review. Clin Infect Dis 1994;19:1067-70.
40. Gass R, Fisher J, Badesch D, Zamora M, Weinberg A, Melsness H, et al. Donor-to-host transmission of *Mycoplasma hominis* in lung allograft recipients. Clin Infect Dis 1996;22:567-8.
41. Bonilla HF, Chenoweth CE, Tully JG, Blythe LK, Robertson J, Ognenovski VM, et al. *Mycoplasma felis* septic arthritis in a patient with hypogammaglobulinemia. Clin Infect Dis. In press.
42. Yechouron A, Lefebre J, Robson HG, Rose DL, Tully JG. Fatal septicemia due to *Mycoplasma arginini*: a new human zoonosis. Clin Infect Dis 1992;15:434-8.
43. Baseman JB. The cytadhesins of *Mycoplasma pneumoniae* and *M. genitalium*. In: Rottem S, Kahane I, editors. Subcellular biochemistry. New York: Plenum Press, 1993;243-59.
44. Kirchhoff H, Rosegarten R, Lotz W, Fischer M, Lopatta D. Flask-shaped mycoplasmas: properties and pathogenicity for man and animals. Isr J Med Sci 1984;10:848-53.
45. Gobel U, Speth V, Bredt W. Filamentous structures in adherent *Mycoplasma pneumoniae* cells treated with nonionic detergents. J Cell Biol 1981;91:537-43.
46. Krause DC. *Mycoplasma pneumoniae* cytadherence: unravelling the tie that binds. Mol Microbiol 1996;20:247-53.
47. Su CJ, Tryon VV, Baseman JB. Cloning and sequence analysis of cytadhesin gene (P1) from *Mycoplasma pneumoniae*. Infect Immun 1987;55:3023-9.
48. Dallo SF, Chavoya A, Baseman JB. Characterization of the gene for a 30-kilodalton adhesin-related protein of *Mycoplasma pneumoniae*. Infect Immun 1990;58:4163-5.
49. Hu PC, Schaper U, Collier AM, Clyde WA, Horikawa M, Huang YS, et al. A *Mycoplasma genitalium* protein resembling the *Mycoplasma pneumoniae* attachment protein. Infect Immun 1987;55:1126-31.
50. Reddy SP, Rasmussen WG, Baseman JB. Molecular cloning and characterization of an adherence-related operon of *Mycoplasma genitalium*. J Bacteriol 1995;177:5943-51.
51. Inamine JM, Loechel S, Gilbert AM, Barile MF, Hu PC. Nucleotide sequence of the MgPa (*mgp*) operon of *Mycoplasma genitalium* and comparison to the P1 (*mpp*) operon of *Mycoplasma pneumoniae*. Gene 1989;82:259-67.
52. Goulet M, Dular R, Tully JG, Billowes G, Kasatiya S. Isolation of *Mycoplasma pneumoniae* from the human urogenital tract. J Clin Microbiol 1995;33:2823-5.
53. Reddy SP, Rasmussen WG, Baseman, JB. Isolation and characterization of transposon Tn *4001*-generated, cytadherence-deficient transformants of *Mycoplasma pneumoniae* and *Mycoplasma genitalium*. FEMS Immunol Med Microbiol 1996;15:199-211.
54. Su CJ, Chavoya A, Baseman JB. Regions of *Mycoplasma pneumoniae* cytadhesin P1 structural gene exist as multiple copies. Infect Immun 1988;56:3157-61.
55. Dallo SF, Baseman JB. Adhesin gene of *Mycoplasma genitalium* exists as multiple copies. Microb Pathog 1991;10:475-80.
56. Peterson SN, Bailey CC, Jensen JS, Borre MB, King ES, Bott KF, et al. Characterization of repetitive DNA in the *Mycoplasma genitalium* genome: possible role in the generation of antigenic variation. Proc Natl Acad Sci USA 1995;92:11829-33.
57. Dallo SF, Horton JR, Su CJ, Baseman JB. Restriction fragment length polymorphism in the cytadhesin P1 gene of human clinical isolates of *Mycoplasma pneumoniae*. Infect Immun 1990;58:2017-20.
58. Su CJ, Chavoya A, Dallo SF, Baseman JB. Sequence divergency of the cytadhesin gene of *Mycoplasma pneumoniae*. Infect Immun 1990;58:2669-74.
59. Su CJ, Dallo SF, Baseman JB. Possible origin of sequence divergence in the P1 cytadhesin gene of *Mycoplasma pneumoniae*. Infect Immun 1993;61:816-22.
60. Dallo SF, Su CJ, Horton JR, Baseman JB. Identification of P1 gene domain containing epitope(s) mediating *Mycoplasma pneumoniae* cytadherence. J Exp Med 1988;167:718-23.
61. Gerstenecker B, Jacobs E. Topographical mapping of the P1-adhesin *of Mycoplasma pneumoniae* with adherence-inhibiting monoclonal antibodies. J Gen Microbiol 1990;136:471-6.
62. Dallo SF, Lazzell AL, Chavoya A, Reddy SP, Baseman JB. Biofunctional domains of the *Mycoplasma pneumoniae* P30 adhesin. Infect Immun 1996;64:2595-601.
63. Layh-Schmitt G, Hilbert H, Pirkl E. A spontaneous hemadsorption-negative mutant of *Mycoplasma pneumoniae* exhibits a truncated adhesin-related 30-kilodalton protein and lacks the cytadherence-accessory protein HMW1. J Bacteriol 1995;177:843-6.
64. Reddy SP, Rasmussen WG, Baseman JB. Correlations between *Mycoplasma pneumoniae* sensitivity to cyclosporin A and cyclophilin-mediated regulation of mycoplasma cytadherence. Microb Pathog 1995;20:155-69.

65. Biberfeld G. Infection sequelae and autoimmune reactions in *Mycoplasma pneumoniae* infection. In: Razin S, Barile MF, editors. The Mycoplasmas Vol. IV. New York: Academic Press, 1985:293-311.
66. Cunningham MW. Molecular mimicry: bacterial antigen mimicry. In: Bona CA, Siminovitch K, Theofilopoulos AN, Zanetti M, editors. The pathology of autoimmunity. New York: Harwood Academic Publishers, 1993:245-56.
67. Bisset LR. The *Mycoplasma genitalium* adhesin protein and several human class II MHC proteins exhibit sequence homology: possible ramifications for the development of autoimmunity. Autoimmunity 1992;14:167-8.
68. Root-Bernstein RS, Hobbs SH. Homologies between mycoplasma adhesion peptide, CD4 and class II MHC proteins: a possible mechanism for HIV-mycoplasma synergism in AIDS. Res Immunol 1991;142:519-23.
69. Cole BC. Mycoplasma interactions with the immune system:implications for disease pathology. ASM News 1996;62:471-5.
70. Rawadi G, Roman-Roman S. Mycoplasma membrane lipoproteins induce proinflammatory cytokines by a mechanism distinct from that of lipopolysaccharide. Infect Immun 1996;64:637-43.
71. Theiss P, Karpas A, Wise KS. Antigenic topology of the P29 surface lipoprotein of *Mycoplasma fermentans*: differential display of epitopes results in high-frequency phase variation. Infect Immun 1996;64:1800-9.
72. Baseman JB, Lange M, Criscimagna NL, Girón JA, Thomas CA. Interplay between mycoplasmas and host target cells. Microb Pathog 1995;19:105-16.
73. Girón JA, Lange M, Baseman JB. Adherence, fibronectin binding, and induction of cytoskeleton reorganization in cultured human cells by *Mycoplasma penetrans*. Infect Immun 1996;64:197-208.
74. Blanchard A, Montagnier L. AIDS-associated mycoplasmas. Annu Rev Microbiol 1994;48:687-712.
75. Wang RY-H, Shih J W-K, Weiss SH, Grandinetti T, Pierce PF, Lange M, et al. *Mycoplasma penetrans* infection in male homosexuals with AIDS: high seroprevalence and association with Kaposi's Sarcoma. Clin Infect Dis 1993;17:724-9.
76. Grau O, Slizewicz B, Tuppin P, Launay V, Bourgeois E, Sagot N, et al. Association of *Mycoplasma penetrans* with human immunodeficiency virus infection. J Infect Dis 1995;172:672-81.
77. Paton GR, Jacobs JP, Perkins FT. Chromosome changes in human diploid-cell cultures infected with *Mycoplasma*. Nature 1966;207:43-5.
78. Macpherson I, Russell W. Transformations in hamster cells mediated by mycoplasmas. Nature 1966;210:1343-5.
79. Kangro HO, Chong SKF, Hardiman A, Heath RB, Walker-Smith JA. A prospective study of viral and mycoplasma infections in chronic inflammatory bowel disease. Gastroenterology 1990;98:549-53.
